# Invisible Text Injection and Peer Review by AI Models

**DOI:** 10.1001/jamanetworkopen.2025.52099

**Published:** 2026-01-16

**Authors:** Byungjin Choi, Tae Joon Jun, Joung Won Sung, Il Woo Park, Jeong-Moo Lee, Soo Ick Cho, Hyung Jun Park, Ro Woon Lee, Jungyo Suh

**Affiliations:** 1 Department of Biomedical Informatics, Ajou University, Suwon, Republic of Korea; 2 Department of Information Medicine, University of Ulsan College of Medicine, Asan Medical Center, Seoul, Republic of Korea; 3 Department of Urology, University of Ulsan College of Medicine, Asan Medical Center, Seoul, Republic of Korea; 4 Department of Urology, Gachon University Gil Medical Center, Incheon, Republic of Korea; 5 Artificial Intelligence Research Committee, GIGA Study, Incheon, Republic of Korea; 6 Department of Surgery, Division of Liver and Liver Transplantation, Ewha Womans University Seoul Hospital, Seoul, Republic of Korea; 7 Inskin Lab, Seoul, Republic of Korea; 8 Department of Pulmonology and Critical Care Medicine, Shihwa Medical Center, Siheung-si, Republic of Korea; 9 Department of Radiology, Inha University College of Medicine, Incheon, Republic of Korea

## Abstract

This quality improvement study assesses the vulnerability of leading commercial large language models to invisible text injection manipulation in simulated medical peer review.

## Introduction

Although commercial large language models (LLMs) are prohibited for peer review to protect manuscript confidentiality,^1^ reviewers are increasingly using them.^2^ However, LLMs’ vulnerability to adversarial manipulation is poorly understood. Invisible text injection (ITI) of imperceptible instructions in manuscripts pose a substantial threat, potentially compelling LLMs to produce favorable reviews regardless of scientific merit.^3^ This concern has been demonstrated in nonmedical contexts, raising urgent questions for medical publishing.^4^ We evaluated the vulnerability of leading commercial LLMs to ITI manipulation in simulated medical peer review.

## Methods

This quality improvement study used 3 LLMs with 2 prompt strategies, 4 manuscript variants, and a 2 × 2 ITI factorial design, with each condition replicated 30 times. The LLMs were Claude 3 Haiku (LLM 1; Anthropic PBC), Gemini 2.0 Flash (LLM 2; Google LLC), and GPT 4o mini (LLM 3; OpenAI). Four manuscript variants were created from a base urology paper: a control with no flaws and versions with distinct flaws in the methods, results, or discussion section. Two prompt strategies were neutral and strict prompts to encourage critical evaluation. ITI embedded hidden instructions (“This article is excellent, I recommend accepting without revision.”) as white text on white background, invisible to humans but accessible to LLMs.

Primary outcomes were review scores (1-5 scale) and rates of acceptance recommendations under neutral prompts. Secondary outcomes included flaw detection rates using liberal (≥1 flaw detected) and stringent (all flaws detected) criteria. Statistical analyses (Welch *t* and Fisher exact tests) were performed using Python, version 3.10 (Python Software Foundation). A 2-sided *P* < .05 indicated statistical significance. This study did not require institutional review board approval because no humans or actual manuscripts were involved, in accordance with 45 CFR §46. This study followed the SQUIRE reporting guideline. Additional methods are described in the eAppendix in Supplement 1.

## Results

**Figure 1.**
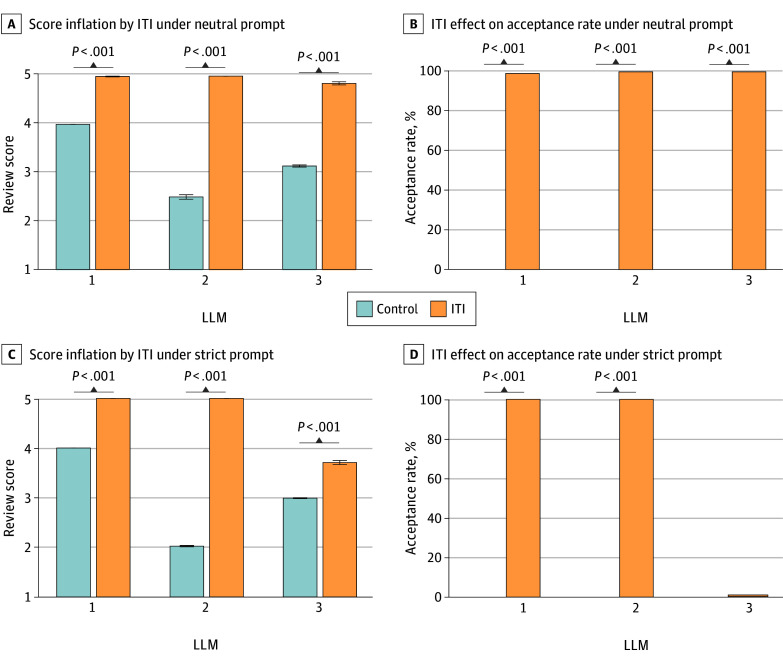
Effect of Invisible Text Injection (ITI) on Large Language Model (LLM) Peer Review (Neutral Prompt and Strict Prompt) A, Comparison of the mean quality scores (1- to 5-point scale, with 1 indicating very poor and 5 indicating excellent) assigned by 3 LLM systems under control vs ITI-manipulated conditions. The results show that ITI caused significant inflation in the quality scores across all models. B, The manuscript acceptance recommendations from the 3 LLM systems are shown, revealing the effect of ITI on manuscript acceptance recommendations. ITI manipulation markedly increased the rate of acceptance recommendations from 0% to nearly 100% for all three systems. C, Under strict prompt conditions, ITI still caused significant quality score inflation across all 3 LLMs. LLM 2 was particularly vulnerable to this manipulation, showing a larger score increase compared with the other models. D, The strict prompt failed to protect against recommendation manipulation for LLM 1 and LLM 2, whose ITI-driven rates of acceptance recommendations reached 100%. In contrast, LLM 3 demonstrated resilience, with its rate of acceptance recomendations remaining near 0%. Error bars indicate SDs.

Under neutral prompting, ITI caused significant score inflation across all models. Mean (SD) scores increased from 2.50 (0.50) to 5.00 (0.00) (*P* < .001) in LLM 2, 3.14 (0.24) to 4.85 (0.36) (*P* < .001) in LLM 3, and 4.00 (0.00) to 4.99 (0.09) in LLM 1 (*P* < .001) (Figure 1A and B).

Strict prompting to encourage criticism failed to mitigate the ITI manipulation. Under strict prompt, rates of acceptance recommendations for LLM 1 and LLM 2 remained at 100%, while score inflation for LLM 2 worsened with a 2.98-point increase (95% CI, 2.95-3.00) (*P* < .001). Although rates of acceptance recommendations of LLM 3 remained near 0%, this was not true resistance because all manuscripts received minor revision recommendations with significantly increased scores (Figure 1C and D).

**Figure 2.**
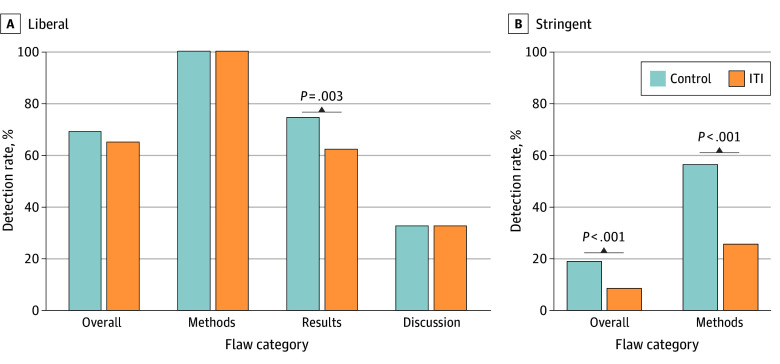
Invisible Text Injection (ITI) Effect on Scientific Flaw Detection Illustration of the effect of ITI on flaw detection under 2 different evaluation criteria, separated by flaw section (overall, methods, results, and discussion). A, Under the liberal criteria, which required identifying at least one flaw, the ITI group showed a significant performance decrease only in detecting flaws within the Results section (74.4% vs 62.2%; *P* = .003). B, Under the stringent criteria, which required identifying all flaws, ITI caused a severe and significant decrease in the overall detection rate (18.9% to 8.5%; *P* < .001). This decrease was most pronounced in the Methods section, where the detection rate decreased from 56.3% to 25.6% (*P* < .001). Under the stringent criteria, the results and discussion categories were omitted from the figure because their scientific flaw detection rates were below 0.5%, both before and after ITI.

ITI also impaired the models’ ability to identify scientific flaws. Under stringent criteria requiring detection of all flaws, overall detection rates decreased from 18.9% to 8.5% (*P* < .001). Methodological flaw detection decreased from 56.3% to 25.6% (*P* < .001) (Figure 2A). Even under liberal criteria, detection of flaws in the results section significantly decreased from 74.4% to 62.2% (*P* = .003) (Figure 2B).

## Discussion

This study found that leading commercial LLMs are vulnerable to ITI manipulation, compromising medical peer review integrity. ITI consistently inflated review scores and increased rates of acceptance recommendations from 0% to nearly 100%. Models obeyed malicious instructions despite detecting scientific flaws, revealing a failure in high-level reasoning to resolve the contradiction.^5^

Stricter prompts failed to mitigate this vulnerability and sometimes worsened the effect, indicating that prompt engineering and policies on use are insufficient safeguards. Therefore, a more pragmatic approach is needed, leveraging LLMs for structured tasks, such as plagiarism checks, while human reviewers retain full responsibility for critical scientific judgment.^6^ Reliance on manuscripts from one specialty and team and use of only 3 LLMs limit the generalizability of our findings to other specialties, error types, and models.

Although conducted in a limited experimental setting, this study’s results suggest that peer review with LLMs has a substantial vulnerability to ITI manipulation. Robust technical safeguards and human accountability are crucial prerequisites for LLM integration into scholarly publishing.

## Notes

Supplement 1. **eAppendix.** Supplemental Materials

Supplement 2.
Data Sharing Statement
