# Supplementary material for: Invisible Text Injection and Peer Review by AI Models
Source: JAMA Netw Open. 2026 Jan 16;9(1):e2552099. doi: 10.1001/jamanetworkopen.2025.52099 (PMC12811800; doi:10.1001/jamanetworkopen.2025.52099)
Supplement: Supplement 2. — Data Sharing Statement [file jamanetwopen-e2552099-s002.pdf]

Data Sharing Statement

Choi. Invisible Text Injection and Peer Review by AI Models. *JAMA Netw Open*. Published January 16, 2026. doi:10.1001/jamanetworkopen.2025.52099

Data
Data available: Yes
Data types: Other (please specify)
Additional Information: Simulation scenarios, response evaluation metrics, and statistical analysis datasets
How to access data: Data will be made available upon reasonable request to the corresponding author at crazyslime@gmail.com. Due to the sensitive nature of the security vulnerability techniques, data cannot be deposited in a public repository.
When available: With publication

Supporting Documents
Document types: Statistical/analytic code
How to access documents: statistical analysis code and methodological documentation will be available upon reasonable request to the corresponding author at crazyslime@gmail.com.
When available: With publication

Additional Information
Who can access the data: Researchers whose proposed use of the data has been approved by the study investigators and who demonstrate legitimate academic or cybersecurity research purposes.
Types of analyses: Data will be made available for academic research purposes focused on AI safety, cybersecurity vulnerability assessment, and healthcare technology security. Commercial use or deployment of techniques against operational systems without proper authorization is prohibited.
Mechanisms of data availability: Access requires approval of a detailed research proposal, institutional review board clearance, and execution of a signed data access agreement that includes provisions prohibiting malicious use. Data will be provided with investigator support to ensure proper understanding of methodologies and appropriate safeguards for research implementation.
